# *Leishmania* infection modulates beta-1 integrin activation and alters the kinetics of monocyte spreading over fibronectin

**DOI:** 10.1038/srep12862

**Published:** 2015-08-07

**Authors:** Cláudio Pereira Figueira, Djalma Gomes Ferrão Carvalhal, Rafaela Andrade Almeida, Micely d’ El-Rei Hermida, Dominique Touchard, Phillipe Robert, Anne Pierres, Pierre Bongrand, Washington LC dos-Santos

**Affiliations:** 1 Fundação Oswaldo Cruz-Bahia, Centro de Pesquisas Gonçalo Moniz, Brazilian Ministry of Health, Salvador, Brazil; 2 Universidade do Estado da Bahia, Salvador, BA, Brazil; 3 Laboratoire Adhésion Cellulaire et Inflammation, Parc Scientifique de Luminy, Aix-Marseille Université, Marseille, France

## Abstract

Contact with *Leishmania* leads to a decreases in mononuclear phagocyte adherence to connective tissue. In this work, we studied the early stages of bond formation between VLA4 and fibronectin, measured the kinetics of membrane alignment and the monocyte cytoplasm spreading area over a fibronectin-coated surface, and studied the expression of high affinity integrin epitope in uninfected and *Leishmania*-infected human monocytes. Our results show that the initial VLA4-mediated interaction of *Leishmania*-infected monocyte with a fibronectin-coated surface is preserved, however, the later stage, leukocyte spreading over the substrate is abrogated in *Leishmania*-infected cells. The median of spreading area was 72 [55–89] μm^2^ for uninfected and 41 [34–51] μm^2^ for *Leishmania*-infected monocyte. This cytoplasm spread was inhibited using an anti-VLA4 blocking antibody. After the initial contact with the fibronectrin-coated surface, uninfected monocyte quickly spread the cytoplasm at a 15 μm^2^ s^−1^ ratio whilst *Leishmania*-infected monocytes only made small contacts at a 5.5 μm^2^ s^−1^ ratio. The expression of high affinity epitope by VLA4 (from 39 ± 21% to 14 ± 3%); and LFA1 (from 37 ± 32% to 18 ± 16%) molecules was reduced in *Leishmania*-infected monocytes. These changes in phagocyte function may be important for parasite dissemination and distribution of lesions in leishmaniasis.

Leishmaniasis is a disease caused by intracellular protozoa of the genus *Leishmania*. Infected sand flies transmit the disease through the skin during blood feeding. Once inoculated into the skin, *Leishmania* infects mononuclear phagocytes. The infected cells may remain at the inoculation site or disseminate through the body, causing lesions in the skin, mucosae or internal organs^1^,^2^,^3^. The disease is characterized by skin and mucosal ulcers or by fever, emaciation, hepatosplenomegaly, hypersplenism, anemia, thrombocytopenia and increased susceptibility to bacterial infections, leading to death^4^.

The mechanisms that control *Leishmania* dissemination through different host tissues are poorly understood. However, evidence suggests that *Leishmania* infection and the parasite burden modulate the migratory capability of mononuclear phagocytes^5^,^6^. In previous studies, we showed that infection with different *Leishmania* species (*L. amazonensis*, *L. braziliensis* or *L. infantum*) impairs the adherence of monocytes and macrophages to connective tissue^7^. Such impairment in leukocyte adhesion is due to interference with integrin function^5^. For example, the inhibitory effect of *Leishmania* infection on inflammatory macrophage adherence to fibronectin is reversed by replacement of the Ca^++^ and Mg^++^ present in the medium with Mn^++^, which causes signaling-independent integrin activation^5^. Furthermore, infection with *Leishmania* downregulates the expression of the genes encoding the chemokine receptors CCR4 and CCR5 in murine inflammatory macrophages and the genes encoding CCR2 and CCR5 in murine dendritic cells^5^,^6^. In addition, *Leishmania* infection leads to decreased dendritic cell migration in response to the chemokines CCL2 and CCL3 in murine dendritic cells^6^. The function of VLA4, a β1 integrin involved in leukocyte adhesion to fibronectin, is modulated by *Leishmania* infection^5^. This molecule may be present on the leukocyte surface in different conformations, and it mediates rolling or firm adherence of the cell to the substrate^8^. When macrophages adhere firmly to the substrate, they spread extensively. This spreading stabilizes the adherence and allows cell haptotaxis toward increasing chemokine concentrations^9^. Hence, coordinated VLA4 activation is crucial for cell emigration or retention in the tissues.

In this work, we expand the observations of our previous studies on the impairment of *Leishmania*-infected macrophage adhesion to connective tissue. We examine the effect of *Leishmania* infection on the rolling and spreading of infected monocytes over fibronectin. We used a flow chamber and applied an algorithm to measure different parameters of monocyte rolling. The kinetics of monocyte spreading over fibronectin was examined by interference reflection microscopy (IRM), and the spreading area was estimated by morphometric analysis using scanning electron microscopy. Furthermore, we used a reporter antibody to study the affinity state of the VLA4 expressed by infected and uninfected monocytes.

## Results

### Rolling of *Leishmania*-infected monocytes on fibronectin

**Figure 1:**
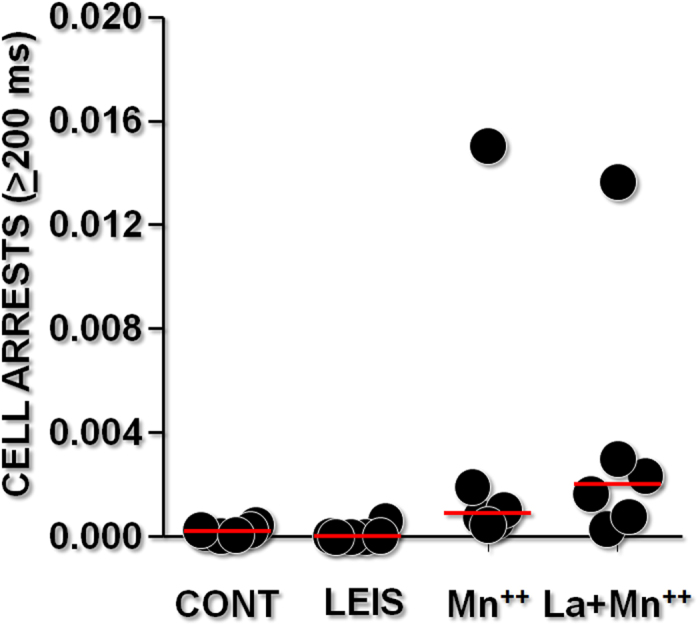
Monocyte adhesion under flow. Monocytes cultured alone or with *Leishmania* were driven along fibronectin-coated surfaces in a laminar flow chamber in medium alone or in medium containing 10 mM MnCl_2_. The trajectories of the individual cells were monitored for 2 min for quantitative determination of the number of total detectable arrests with durations between ∼200 ms and 2 min.

To identify *Leishmania*-induced changes in the VLA4-mediated rolling of monocytes, we used an *in vitro* model of laminar flow to compare this adhesion step in uninfected and *Leishmania*-infected cells. *Leishmania*-infected monocytes displayed numerous transient arrests, with a frequency comparable with that found for uninfected monocytes (Fig. 1). After Mn^++^ stimulation, the frequency of arrests increased in both uninfected and *Leishmania*-infected monocytes. Taken together, these results demonstrate that *Leishmania* infection did not interfere with VLA4-mediated monocyte rolling or initial binding to fibronectin.

### Spreading of *Leishmania*-infected human monocytes on fibronectin

**Figure 2:**
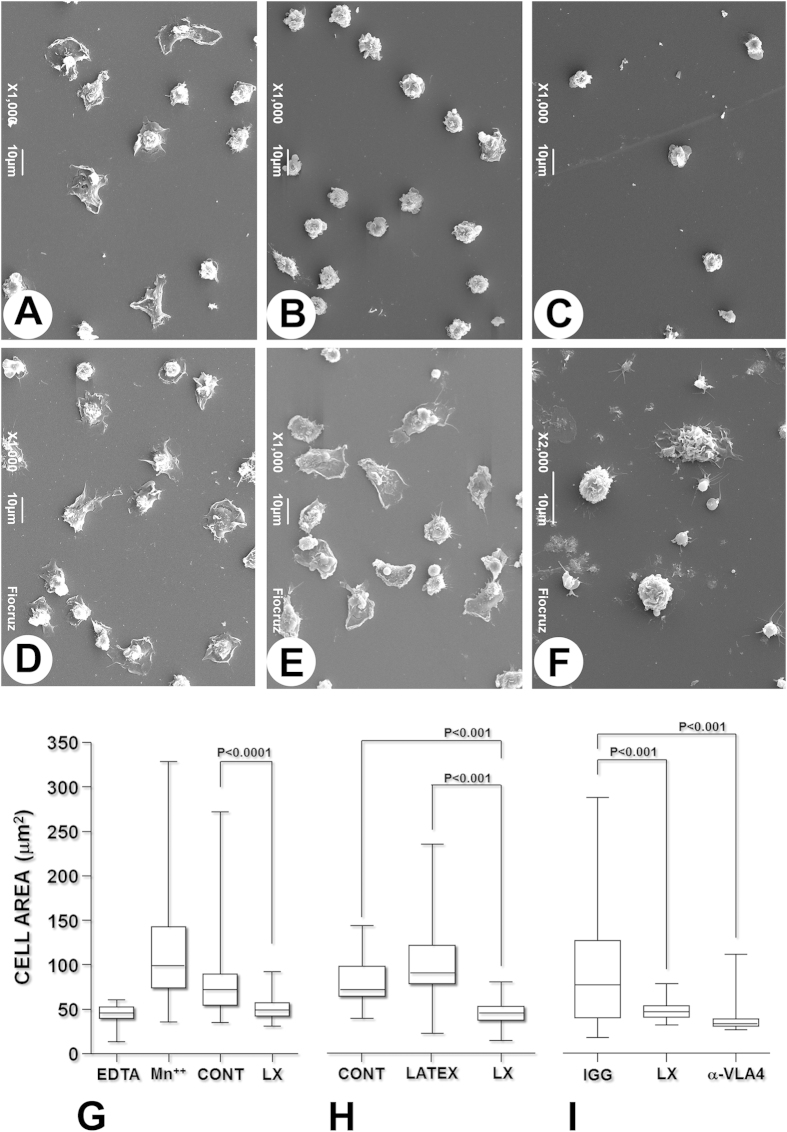
Spreading of human monocyte cytoplasm on fibronectin after *Leishmania* infection. Peripheral blood monocytes were cultured with medium alone (**A**,**C**,**D**,**F**) or with medium containing *Leishmania* (**B**) or 3 μM latex beads (**E**) for 18 h. The cells were then allowed to adhere for 1 h to fibronectin-coated coverslips: (**A**) – Uninfected (control) monocytes; (**B**) – monocytes cultured with *Leishmania*; (**C**) – uninfected cells treated with 0.5 mM EDTA; (**D**) – uninfected cells treated with 0.5 mM MnCl_2_; (**E**) – uninfected cells cultured with latex beads; (**F**) – uninfected cells treated with anti-VLA4 antibody. The graph shows the area over which the cytoplasm of the monocytes had spread after the various treatments. The data are representative of six (**G**) and two (**H**,**I**) different experiments (Kruskal-Wallis test).

Because we did not observe changes in the initial step (rolling) of infected monocyte adherence to fibronectin, we used SEM to compare the cytoplasmic spreading of uninfected and *Leishmania*-infected human monocytes on fibronectin. Most of the monocytes cultured with medium alone displayed a flattened cell phenotype with extensive cytoplasmic spreading and irregular edges (Fig. 2A). Uninfected monocytes cultured with medium alone or cultured with 3 μm latex beads showed similar phenotypes (Fig. 2E), as did uninfected monocytes treated with Mn^++^ just before the adhesion assay (used as a control for high-affinity adhesion, Fig. 2D). On the other hand, most of the monocytes cultured with *Leishmania* had a rounded morphology with low levels of cytoplasmic spreading (Fig. 2B), which was similar to the morphology observed when the monocytes were treated with EDTA before the adhesion assay (used as a negative control for cytoplasmic spreading, Fig. 2C). The spread area (μm^2^) of the monocyte cytoplasm, 72 [55–89] (median [lower and upper quartiles]), was larger for the monocytes cultured with medium alone than for the monocytes cultured with *Leishmania* (49 [43–57]; Mann-Whitney test, P < 0.0001, Fig. 2G).

Treatment with the anti-α4 chain 9F10 anti-VLA4 blocking antibody inhibited monocyte spreading to the same extent as exposure of the cells to *Leishmania* (Fig. 2F,I).

**Figure 3:**
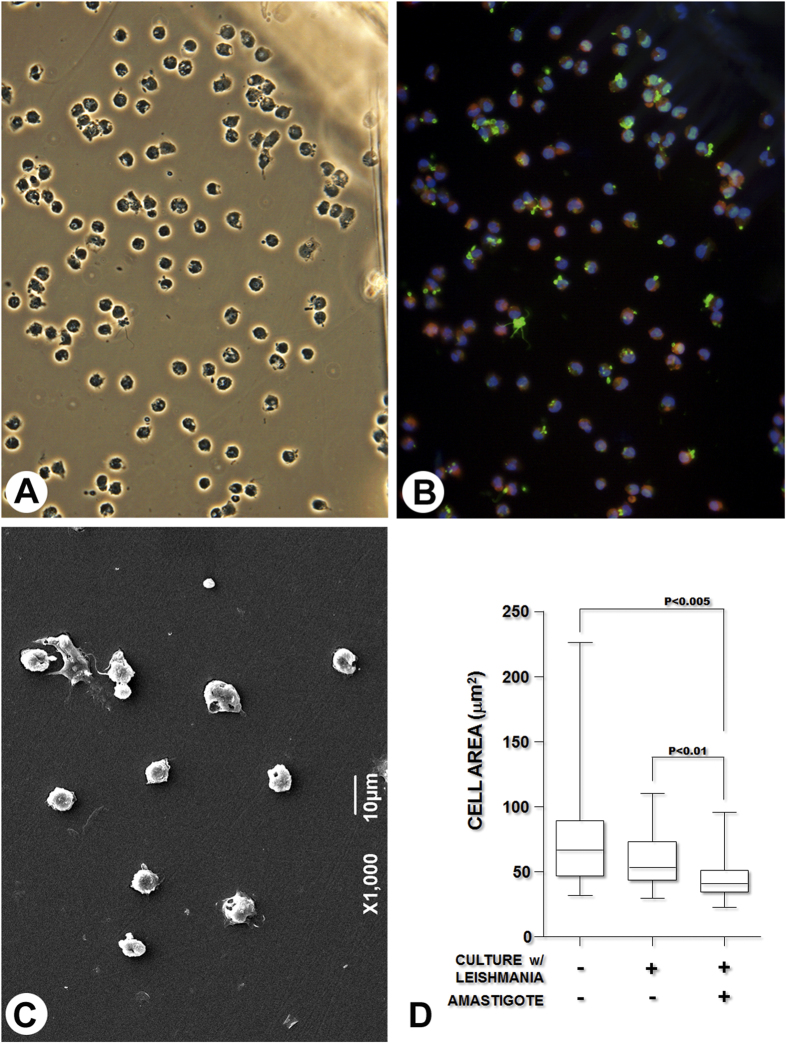
Correlative analysis of the cytoplasmic spreading of human monocytes cultured with medium alone or with medium containing *Leishmania*. The cells were allowed to adhere to precoated Aclar coverslips containing a grid to allow the identification of the same cell in immunofluorescence and scanning electron microscopy preparations as described in Materials and Methods. (**A**) – Phase contrast image showing the cells adhered to the Aclar membrane with part of the grid used as a reference. (**B**) – Immunofluorescence image showing uninfected human monocytes or monocytes containing *Leishmania* amastigotes (blue = DAPI; red = phalloidin; green = *Leishmania* amastigotes). (**C**) – Scanning electron microscopy image showing the area corresponding to the insert in part **B**. (**D**) – Graphical representation of the area of cytoplasmic spread of uninfected monocytes cultured with medium alone (without contact with *Leishmania*), uninfected monocytes cultured with *Leishmania* or monocytes containing amastigotes (Kruskal-Wallis test).

To determine whether monocyte infection with *Leishmania* was specifically necessary for the inhibition of cytoplasmic spreading, we combined SEM with amastigote identification in the interior of the monocytes using a technique described by Jiménez and colleagues (2010) (Fig. 3)^10^. The area of the cytoplasmic spread of the amastigote-containing monocytes (41 [34–51]) was smaller than that observed for monocytes cultured with medium alone (66 [47–89], P < 0.05) or that for monocytes that had been cultured with the parasites but did not contain amastigotes (53 [44–73], P < 0.05, Fig. 3D).

### Monocyte-*Leishmania* contact and leukocyte adherence to connective matrix components

**Figure 4:**
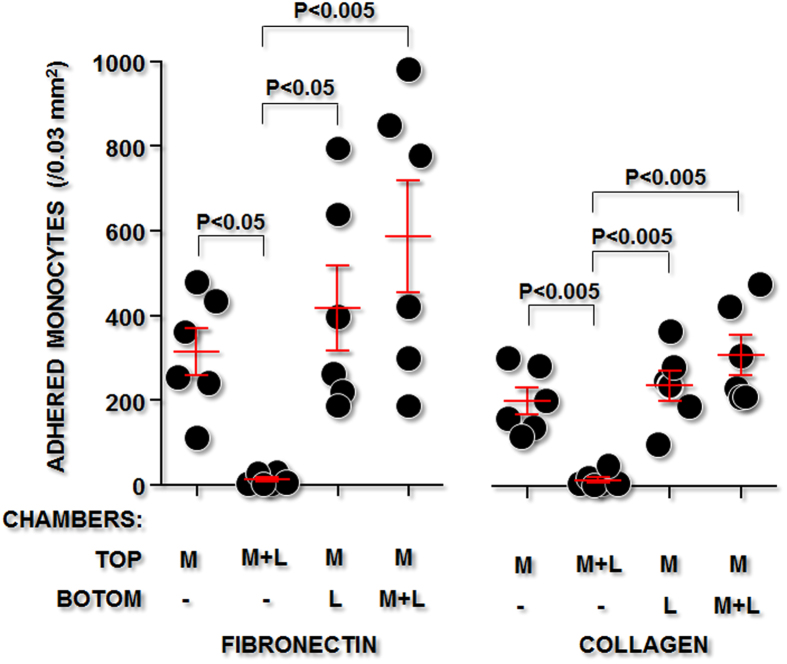
The effect of soluble factors from *Leishmania* on the adhesion capacity of human monocytes. Human monocytes were cultured in the top chamber of transwell modules either alone (M) or with *Leishmania* (M + L). The bottom chamber contained medium, *Leishmania* (L), or macrophages and *Leishmania* (M + L). After 24 h, the cells in the top chamber were collected and subjected to the adhesion assay on surfaces coated with fibronectin or collagen.

To confirm that infection—and not soluble substances released by the *Leishmania* or by the infected leukocytes—would interfere with monocyte adherence to connective matrix components, we performed an adhesion assay using monocytes cultured in contact with the parasites or separated from them by a permeable membrane in transwell chambers. Only monocytes that were cultured in contact with *Leishmania* displayed decreased adherence to fibronectin or to collagen (Fig. 4). The monocytes in contact with the parasite showed a 96.2% decrease in adherence to fibronectin and a 92.5% decrease in adherence to collagen in comparison with the uninfected control monocytes cultured with medium alone. Monocytes cultured in a chamber separate from the one containing the *Leishmania* showed no change in adherence to the connective matrix components (Fig. 4).

### Kinetics of cell–surface contact between *Leishmania*-infected monocytes and fibronectin

**Figure 5:**
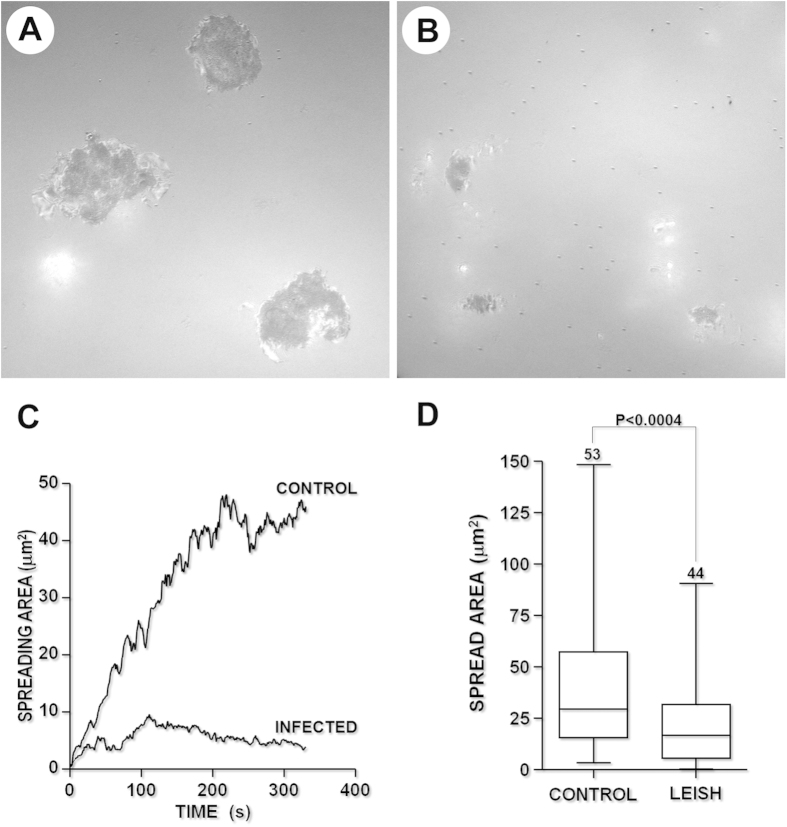
Kinetics of the cytoplasmic spreading of uninfected (**A**) or *Leishmania*-infected (**B**) human monocytes on a fibronectin-coated surface, estimated by IRM. The data on the kinetics of cytoplasmic spreading are shown in graph **C**, which shows uninfected monocytes (CONTROL) and monocytes cultured with *Leishmania* (INFECTED). (**D**): Estimate of the cell contact area obtained from random fields. The graph is representative of four independent experiments. Scale bar, 5 μm.

Because we showed that the spreading capacity was impaired in *Leishmania*-infected monocytes, we used IRM to compare the kinetics of membrane contact of infected and uninfected monocytes with fibronectin-coated coverslips. Upon contact with the surface, uninfected monocytes quickly began to spread and initiated stable adhesion (Fig. 5A and supplementary video S1), while the *Leishmania*-infected monocytes contacted the substrate only through small and unstable adhesion areas (Fig. 5B and supplementary video S2). The uninfected monocyte-substrate contact area linearly increased at a rate of approximately 15 μm^2^ s^−1^, which was three-fold higher than the rate of *Leishmania*-infected monocytes (5.5 μm^2^ s^−1^, Fig. 5C). The final area of cytoplasmic spread of *Leishmania*-infected monocytes (22.8 μm^2^) was smaller than that of the uninfected cells (67.98 μm^2^, P < 0.0004, Fig. 5D).

### Integrin activation on *Leishmania*-infected human monocytes

**Figure 6:**
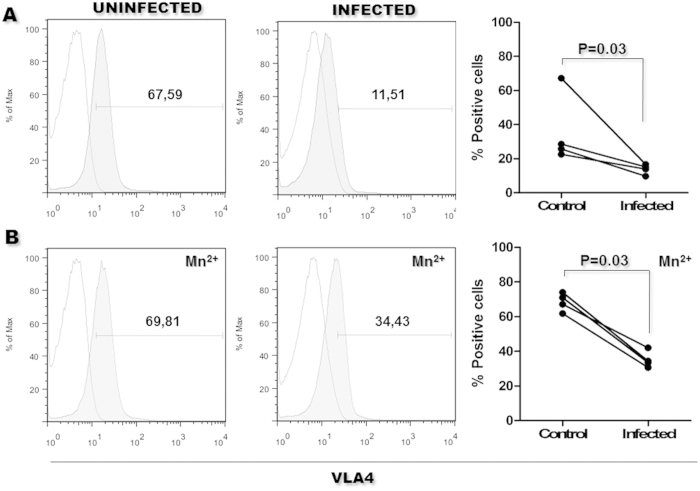
High-affinity VLA-4 epitope expression on infected monocytes. For single-color staining, 1 × 10^6^ monocytes were cultivated in medium alone or in medium containing *Leishmania*. The cells were resuspended in medium alone (**A**) or in medium containing Mn^++^ (**B**) The cells were then stained with an anti-VLA4 high-affinity epitope antibody and analyzed by flow cytometry. Empty histograms cells incubated with isotype control antibody. Grey histograms cells incubated with the anti-VLA4 high affinity epitope antibody. Scatterplots: Each pair of data connected by line represents an independent experiment using the same batch of monocytes, cultured with medium alone (Control) or with medium containing *Leishmania* (Infected) (Mann-Whitney test).

Integrin molecules may assume different conformational states, which may correlate with their affinity for their ligands^8^,^11^. We estimated the percentage of cells expressing a high-affinity VLA4 epitope in uninfected and *Leishmania*-infected monocytes using a reporter monoclonal antibody (HUTS-4) and flow cytometry. The expression of high-affinity β1-integrin was lower among the *Leishmania*-infected monocytes (14 ±3 %) than among the uninfected monocytes (36 ± 21%, Fig. 6A). Interestingly, even after stimulation with Mn^++^, the expression of the high-affinity VLA4 epitope was lower in the *Leishmania*-infected monocytes (35 ± 5%) than in the uninfected monocytes (68 ± 5%, Fig. 6B).

**Figure 7:**
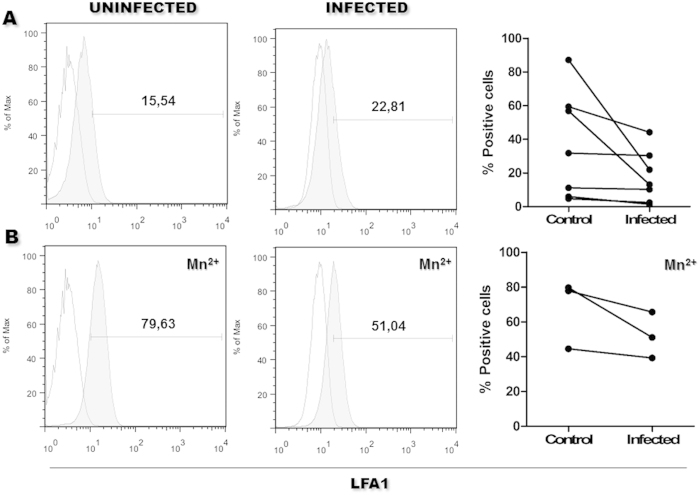
High-affinity LFA1 epitope expression on infected monocytes. For single-color staining, 1 × 10^6^ monocytes were cultured in medium alone or in medium containing *Leishmania*. The cells were resuspended in medium alone (**A**) or in medium containing Mn^++^ (**B**). The cells were then stained with a specific LFA1 high-affinity epitope marker and analyzed by flow cytometry. Empty histograms cells incubated with isotype control antibody. Grey histograms cells incubated with the anti-LFA1 high affinity epitope antibody. Scatterplots: Each pair of data connected by line represents an independent experiment using the same batch of monocytes, cultured with medium alone (Control) or with medium containing *Leishmania* (Infected) (Not significant Mann-Whitney test).

To determine whether this disturbance in the integrin configuration was restricted to VLA4 or was a more generalized effect of *Leishmania* infection, we studied the expression of the high-affinity epitope of LFA1, a beta-2 integrin expressed by leukocytes. The expression of the LFA1 high-affinity epitope was lower in *Leishmania*-infected monocytes (18 ± 16%) than in uninfected monocytes (37 ± 32%, Fig. 7A). Even after stimulation with Mn^++^, the expression of the LFA1 high-affinity epitope remained lower in the *Leishmania*-infected monocytes (52 ± 13%) than in the uninfected monocytes (67 ± 20%); however, this difference was not statistically significant (Fig. 7B).

## Discussion

In previous studies, we demonstrated that co-culture with *Leishmania* inhibits mononuclear phagocyte adhesion to connective tissue and extracellular matrix components^5^,^7^. This decrease in leukocyte adhesion correlated with the parasite burden in these mononuclear phagocytes and was reversed by integrin activation with Mn^++^^5^. There were no consistent changes in adhesion molecules expression in *Leishmania*-infected cells^5^,^7^. These observations suggest that the control of integrin function rather than integrin expression was impaired in infected cells. In this work, we expand our investigation of the effects of *Leishmania* infection on monocyte adhesion. We examined the early stages of VLA4-mediated bond formation between the connective matrix and the cells under conditions of flow, and we observed the late stages of integrin-mediated phagocyte spreading over the substrate. The data presented here show that early bond formation during monocyte interaction with the connective matrix remains unchanged; however, the subsequent cytoplasmic spreading over the extracellular matrix components is inhibited by *Leishmania* infection. We also revealed decreased expression of the high-affinity epitopes of the integrins on infected monocytes and confirmed that infection, and not simply contact with *Leishmania*, is necessary for inducing changes in monocyte adhesion.

VLA4 may be present in different conformations on the leukocyte surface, allowing tethering, rolling or firm adhesion to endothelial cells and matrix components, such as fibronectin, that may be distributed in the connective matrix or on bound to the endothelial cell surface^8^,^12^. We used a flow chamber to investigate whether *Leishmania* infection would change these early contacts mediated by the VLA4 integrin. Flow chambers have been used to mimic leukocyte–endothelial interactions in blood vessels. However, these chambers also provide a highly sensitive means of detecting individual bond formation and rupture when they are operated with a shear rate on the order of 10 s^−1^ in association with an image processing system that can allow the detection of events only a few tens of milliseconds in duration^13^. As shown in this work, human monocytes were able to form low-frequency binding contacts with collagen or fibronectin that allowed the monocytes to roll over the surfaces covered with these connective matrix components. The kinetics of this process was not altered after monocyte co-cultivation with *Leishmania*. Even when Mn^++^ was used to induce integrin activation, both control and infected cells displayed a similar increase in adhesion frequency and in adhesion strengthening. This result suggests that *Leishmania* infection did not affect the capability of integrin to mediate the initial binding to connective matrix components.

In static models of leukocyte adhesion to endothelial cells or to connective tissue components, the initial integrin-mediated binding is followed by progressive spreading of the cell cytoplasm, as shown in our previous work and confirmed in this study^5^,^14^. This step involves an extensive reorganization of leukocyte adhesion receptors and the cellular cytoskeleton to allow close membrane contact with the substrate^15^. The process is triggered upon contact with a small region of the membrane that expands to a large area, and it involves membrane deformability and changes in the conformation/affinity and alignment of adhesion receptors^16^. In this study, we used IRM to monitor these events during adherence of uninfected and *Leishmania*-infected monocytes to fibronectin-coated coverslips. Although the *Leishmania*-infected monocytes developed protrusions of the membrane after the first contact with the substrate, they were not able to spread their cytoplasm over the fibronectin-coated surface; instead, only small contact zones were maintained. This observation was further corroborated by morphometric assessment of the area of the substrate covered by cytoplasm using scanning electron microscopy. Uninfected control cells flattened and exhibited wide and thin cytoplasmic expansion over the fibronectin during the 60 min incubation period. The cytoplasmic spreading was inhibited in the presence of VLA4-blocking antibodies. Most of the *Leishmania*-infected monocytes remained round or emitted only small cytoplasmic projections, appearing similar to those observed in the presence of the anti-VLA4 antibody. Taken together, these observations suggest that although leukocyte membrane deformability may be preserved in *Leishmania*-infected cells, infection interferes with the mechanisms that control integrin function. At least two different processes are involved in integrin-mediated firm adherence of leukocytes to the connective tissue: changes in integrin affinity and integrin aggregation in small clusters at the adhesion points^17^. Both of these processes are potentially affected by *Leishmania* infection: *Leishmania* infection may disrupt lipid raft formation on the leucocyte surface. This change may affect integrin clustering at the focal adhesion points^18^. In this study, using conformation reporter antibodies in flow cytometry, we showed a decrease in expression of the high-affinity VLA4 epitope on the surfaces of *Leishmania*-infected cells.

The mechanisms of affinity regulation and capacity to mediate rolling or firm adhesion differ among integrin families. The lack of the I domain in the VLA4 alpha chain may be responsible for most of these functional differences^8^. However, in this study, we showed that *Leishmania* infection induced lower expression of the high-affinity epitopes of both beta-1 (VLA4) and beta-2 (LFA1) integrins. This observation suggests that a more general impairment in the inside-out pathways of integrin activation is present during *Leishmania* infection. For instance, *Leishmania* infection has an inhibitory effect on a variety of signal transduction pathways potentially affecting integrin function (reviewed by Shio and colleagues in 2012^19^). Furthermore, *Leishmania* infection decreases the gene expression of various chemokine receptors by mice mononuclear phagocytes^5^,^6^. Further studies are necessary to investigate the pathways leading to the changes in integrin affinity reported in this study and whether the capability to form integrin clusters at the cell-substrate interface is also altered in *Leishmania*-infected cells.

*Toxoplasma gondii*, another intracellular protozoan, promotes a similar effect, inhibiting the adherence and cytoplasmic spread of human monocytes over endothelial cell monolayers^20^. The decrease in cytoplasmic spreading after leukocyte contact with *Leishmania* was not observed when the cells were incubated with 3 μm latex particles, which are the same size as the parasite. These data suggest that phagocytosis alone or cell deformation by internalized particles was not sufficient to disturb the function of integrins on the infected cells. In contrast, infection of the monocytes was required for the inhibition of their spreading capacity. Only a non-significant trend toward a decrease in the cytoplasmic area was observed in monocytes that contained no amastigotes after co-culture with *Leishmania*. This observation concurs with the experiments using transwell chambers shown in this study. In these experiments, uninfected monocytes subjected to soluble molecules potentially released by *Leishmania* promastigotes or by *Leishmania-*infected cells had no change in adherence to the connective tissue. Moreover, in a previous study, we showed that, although incubation with live infectious *Leishmania* inhibited inflammatory macrophage adhesion to fibronectin, incubation of leukocytes with killed amastigotes, even at a high parasite-to-macrophage ratio, produced no changes in leukocyte adherence to fibronectin^5^.

Finally, integrins are important for cell migration and homing in different tissues. They are also involved in the cellular signaling that leads to lymphocyte priming and activation. The impairment in leukocyte integrin function shown here may interfere with the course of *Leishmania* infection. In cutaneous leishmaniasis, promastigotes are injected into the skin, where they infect leukocytes. These leukocytes may remain at the injection site or migrate to the draining lymph nodes where they present parasite antigens to T lymphocytes. In a recent study, we showed that dendritic cells migrate less efficiently from an inflammatory site to a draining lymph node after coculture with *Leishmania*^3^. The persistence of these cells at the inoculation site may favor the late development of ulcers in the skin. Both LFA1 and VLA4 participate in assembly and signaling through the immunological synapse during lymphocyte activation. Further studies are necessary to investigate the potential modulatory effect of integrin dysfunction during the disease caused by *L. amazonensis* and other *Leishmania* species capable of changing phagocyte adhesion^7^.

## Materials and Methods

### Ethics statement

The study was conducted in accordance with resolution No. 196/96 of the Brazilian National Health Council. The only human specimen used was peripheral blood monocytes isolated from discarded buffy-coats from undisclosed donors. All the procedures are in accordance with the Helsinki Declation of 1964 and were approved by the Ethics Committee for Research Involving Human Subjects of Centro de Pesquisa Gonçalo Moniz, Fiocruz-BA (Decision No. 262/2012).

### Human monocytes

Peripheral blood monocytes were isolated from healthy volunteers or from buffy coats provided by the Etablissement Français du Sang (EFS) or the Hemocenter of Bahia (HEMOBA). Peripheral blood mononuclear cells were purified by centrifugation over a Ficoll-Paque^TM^ Plus solution (GE Health care, Sweden). Monocytes were obtained using a Percoll (Pharmacia, Sweden) gradient, as previously described^21^, or by negative selection using magnetic cell sorting (Miltenyi Biotec, San Diego, CA, USA). The monocytes were cultured in complete RPMI (Gibco, USA) - RPMI containing 10% fetal bovine serum (Gibco, USA), 50 mg/mL gentamicin (Sigma-Aldrich, USA).

### Parasites and phagocyte infections

Stationary phase *L. amazonensis* (MHOM/BR88/BA-125 or LV79 strain) parasites were grown in Schneider’s insect medium (Sigma-Aldrich, USA) containing 10% fetal bovine serum (Gibco, USA), 50 mg/mL gentamicin at 24 °C. The monocytes were resuspended at 2 × 10^6^/mL in 2 mL of complete RPMI alone or containing *L. amazonensis* or 3-μm-diameter latex beads (Sigma-Aldrich, USA), with ten parasites or particles per leukocyte. The cell suspensions were cultured for 16–18 h at 37 °C with 5% CO_2_ in non-adherent polypropylene tubes, washed with HBSS (Sigma-Aldrich, USA) and utilized in the adhesion assays and flow cytometry experiments. The infection rates were estimated using cytospin preparations stained with Giemsa. In all the experiments, the infection rates were between 75 and 90%.

In some experiments, 2 × 10^6^ monocytes/mL were cultured in the top chamber of a transwell module with a 0.4 μm pore size (Corning, USA). The following preparations were present in the bottom chamber: (1) medium alone; (2) 2 × 10^7^ *Leishmania* promastigotes/mL in complete RPMI; or (3) 2 × 10^6^ monocytes/mL plus 2 × 10^7^ promastigotes/mL in complete RPMI. The cells from the top chamber were used in the adhesion assays.

### Substrate preparation for adhesion assays

The wells of 96-well plates, 12-mm-diameter glass coverslips and 12-mm-diameter Aclar® films (EMS, USA) were covered with collagen (Sigma-Aldrich, USA) or human plasm fibronectin (Sigma-Aldrich, USA) as proposed by Pinheiro and colleagues (2006)^5^.

### Immunofluorescence reaction for *Leishmania* detection

The Aclar films containing adherent formaldehyde-fixed monocytes were washed with HBSS, incubated with HBSS containing bovine serum albumin (BSA) (Sigma-Aldrich, USA) (1%) and Tween-20 (Ludwig Biotec, Brazil) (0.05%) (HBSS-BSA-Tween) for 30 min at RT followed by addition of a polyclonal rabbit anti-*Leishmania* antibody^22^ at a 1:500 dilution in HBSS-BSA-Tween for 2 h at RT. The cells were washed and incubated with an anti-rabbit antibody-Alexa488 (Invitrogen, USA) at a 1:200 dilution in HBSS-BSA-Tween at RT for 1 h. The Aclar films were mounted on a glass slide using Vectashield-DAPI mounting media (Vector, USA). Images of uninfected or amastigote-containing monocytes were recorded using a fluorescence microscope (Olympus BX51, Japan) for correlative analysis. The Aclar films were carefully removed from the slides, fixed in glutaraldehyde (Sigma-Aldrich, USA) (2%) and processed for scanning electron microscopy.

### Monocyte adhesion under laminar flow

The adhesion experiments were performed as described previously^14^. Briefly, the monocytes were subjected to laminar flow through a channel (2 mm width and 0.1 mm height) inserted into a well of a 24-well chamber. The shear rate was ~10 s^−1^; thus, the cells attached to the surface were subjected to a viscous force on the order of 7 pN^23^. Images were acquired with a video camera (HyperHAD; Sony France, Clichy, France) attached to an Olympus IX 50 inverted microscope with a 10× objective set within a closed box maintained at 37 °C. The pixel size was 1 × 1 μm^2^. Sequences, typically 2 min in duration, were digitized and DivX compressed with a Win-TV digitizer (Hauppauge, France) for subsequent analysis. The films were decomposed into frames using ImageJ software (1.48v; NIH, USA). Subsequently, the TrajFSerie program (written in Java) used these tables to create a file containing the trajectory of each cell. This file was processed with Igor software (Wavemetrics, EUA) to detect and measure the duration of the stops of each cell. The numbers of detectable arrests (remaining for 200 ms or longer) and the numbers of permanent arrests (i.e., arrests lasting 2 min) were thus determined.

### Adhesion assays and VLA4-blocking experiments

These analyses were performed in 96-well plates, on 12-mm-diameter glass coverslips (Fisher Scientific, USA) or on 12-mm-diameter Aclar films that were previously labeled on one of their surfaces with a 1 mm grid. The wells of the 96-well plates, the glass coverslips and the Aclar film were coated with human plasma fibronectin (10 μg/mL) for 1 h at 37 °C, washed three times in HBSS and incubated with a 0.1% solution of BSA for 30 min. The BSA solution was discarded, and the monocytes (cultured either alone or with *Leishmania*) were seeded into the wells and incubated for 1 h at 37 °C with 5% CO_2_ and treated as described below. In some experiments, Ca^++^ and Mg^++^ in the medium were replaced by incubating the leukocytes in 1 mM EDTA (Sigma-Aldrich, USA) for 5 min, washing in Ca^++^- and Mg^++^-free HBSS and resuspending the cells in HBSS containing 1% BSA and 0.5 mM MnCl_2_ (Sigma-Aldrich, USA). In some experiments, interactions between VLA4 and fibronectin were blocked by incubating the cells with an anti-CD49d antibody (clone 9F10; BD Pharmingen, USA) at 20 μg/mL for 30 min on ice before they were added to the plate. Control cells were incubated with an isotype-matched antibody (BD Pharmingen, USA).

A total of 8 × 10^4^ cells in 200 μL of medium were dispensed into each well of the 96-well plate. At the end of the incubation, the wells were washed four times with warm HBSS and fixed with 1% glutaraldehyde in warm HBSS.

A total of 5 × 10^5^ cells in 500 μL of medium were dispensed into each well of the 24-well plate containing the glass coverslips. At the end of the incubation, the medium in the wells containing the glass coverslips was carefully replaced with 2.5% glutaraldehyde and 2% freshly prepared formaldehyde, and the coverslips were processed for scanning electron microscopy.

A total of 5 × 10^5^ cells in 500 μL of medium were dispensed into each well of the 24-well plate containing the grid-labeled Aclar films. At the end of the incubation, the cells were fixed by carefully replacing the medium with a freshly prepared 2% formaldehyde solution, incubated overnight at 4 °C and processed for *Leishmania* identification by immunofluorescence followed by scanning electron microscopy.

### Scanning electron microscopy

The coverslips or Aclar films containing attached and fixed monocytes were washed three times with 0.1 M sodium cacodylate (Aldrich-Sigma, USA) (pH 7.4) for 1 h, dehydrated in ethanol and dried using a critical point apparatus (Leica EM CPD030, Austria) with CO_2_. The coverslips were mounted on aluminum stubs, sputter-coated with gold (DESCK IV; Denton Vacuum, USA), and examined using a scanning electron microscope (JSM6394LV; JEOL, Japan) operated at 12 kV. Images of randomly selected fields were collected, and the area of the cell cytoplasm was estimated using ImageJ software (1.48v; NIH, USA). The measurements were made without knowledge regarding the group memberships of each sample.

### Interference Reflection Microscopy Analysis

This technique is used to study the interaction of living cells with a substrate in culture. Aliquots (2 mL) of a cell suspension containing 2 × 10^6^ monocytes alone or 2 × 10^6^ monocytes plus *Leishmania* at a ratio of 2 or 10 promastigotes/monocyte were incubated for 16–18 h at 37 °C with 5% CO_2_. The cells were then washed three times with complete medium by centrifugation at 1500 rpm/7 min. The cell concentration was adjusted to 2.0 × 10^5^ cells/mL, and the cells were transferred into the IRM chamber and dispensed onto coverslips coated with human plasma fibronectin that were previously positioned in the inverted microscope (Axiovert 135 Zeiss, Germany), which was equipped with a heated stage set at 37 °C. Interference reflection microscopy was performed with an antiflex objective (63× magnification, 1.25 NA). To evaluate the spreading kinetics, sequential images of the same field were acquired at a frame rate of 1 per second for 15 min after the addition of the cells to the chamber. Then, at intervals of 15 min, additional images of randomly selected fields were acquired for cell area analysis. Cell adhesion area analysis was performed using Essaiw software^16^.

### Flow cytometry analysis

Uninfected or *Leishmania*-infected monocytes were counted and resuspended in FACS buffer (PBS containing 1% bovine serum albumin and 0.01% sodium azide) and treated with 5% mouse serum plus 5% FBS for 30 min on ice to block non-specific staining. The cells (1−2 × 10^6^) were then incubated with the following fluorescein-conjugated antibodies: HUTS-4 (anti–beta 1 integrin monoclonal antibody, Milli-Mark, UK), mAb24 (anti-beta 2 integrin, Hycult Biotec, USA) or an isotype-matched negative control. All antibody incubations were performed at 4 °C for 20 min and were followed by three washes with FACS buffer. The cells were analyzed on a FACSAria III flow cytometer using Diva software (Becton-Dickinson, USA).

### Expression and analysis of the results

The numerical data are presented in graphical format and represent the absolute values, means, medians, lower and upper quartiles (in brackets), or proportions as stated. For comparisons of the absolute values between two groups, the Mann-Whitney test was used. For comparisons involving more than two groups, the Kruskal-Wallis test followed by Dunn’s test for selected pairs of groups was used in accordance with our experimental design^24^. The level of significance was established at P < 0.05.

## Additional Information

**How to cite this article**: Figueira, C. P. *et al.* *Leishmania* infection modulates beta-1 integrin activation and alters the kinetics of monocyte spreading over fibronectin. *Sci. Rep.* **5**, 12862; doi: 10.1038/srep12862 (2015).

## Supplementary Material

Supplementary Video S1

Supplementary Video S2
